# Traffic-Related Air Pollution and Cognitive Function in a Cohort of Older Men

**DOI:** 10.1289/ehp.1002767

**Published:** 2010-12-20

**Authors:** Melinda C. Power, Marc G. Weisskopf, Stacey E. Alexeeff, Brent A. Coull, Avron Spiro, Joel Schwartz

**Affiliations:** 1 Department of Environmental Health, Harvard School of Public Health, Boston, Massachusetts, USA; 2 Department of Epidemiology, Harvard School of Public Health, Boston, Massachusetts, USA; 3 Department of Biostatistics, Harvard School of Public Health, Boston, Massachusetts, USA; 4 Veterans Affairs Boston Healthcare System, Boston, Massachusetts, USA; 5 Department of Epidemiology, Boston University School of Public Health, Boston, Massachusetts, USA

**Keywords:** aging, black carbon, cognitive dysfunction, epidemiology, particulate matter

## Abstract

**Background:**

Traffic-related particles induce oxidative stress and may exert adverse effects on central nervous system function, which could manifest as cognitive impairment.

**Objective:**

We assessed the association between black carbon (BC), a marker of traffic-related air pollution, and cognition in older men.

**Methods:**

A total of 680 men (mean ± SD, 71 ± 7 years of age) from the U.S. Department of Veterans Affairs Normative Aging Study completed a battery of seven cognitive tests at least once between 1996 and 2007. We assessed long-term exposure to traffic-related air pollution using a validated spatiotemporal land-use regression model for BC.

**Results:**

The association between BC and cognition was nonlinear, and we log-transformed BC estimates for all analyses [ln(BC)]. In a multivariable-adjusted model, for each doubling in BC on the natural scale, the odds of having a Mini-Mental State Examination (MMSE) score ≤ 25 was 1.3 times higher [95% confidence interval (CI), 1.1 to 1.6]. In a multivariable-adjusted model for global cognitive function, which combined scores from the remaining six tests, a doubling of BC was associated with a 0.054 SD lower test score (95% CI, −0.103 to −0.006), an effect size similar to that observed with a difference in age of 1.9 years in our data. We found no evidence of heterogeneity by cognitive test. In sensitivity analyses adjusting for past lead exposure, the association with MMSE scores was similar (odds ratio = 1.3; 95% CI, 1.1 to 1.7), but the association with global cognition was somewhat attenuated (−0.038 per doubling in BC; 95% CI, −0.089 to 0.012).

**Conclusions:**

Ambient traffic-related air pollution was associated with decreased cognitive function in older men.

Declining or comparatively low cognitive function in older adults is associated with loss of independence in activities of daily living (Greiner et al. 1996; McGuire et al. 2006), nursing home admission (Gaugler et al. 2007; Joray et al. 2004), hospitalization (Chodosh et al. 2004), and mortality (Bassuk et al. 2000; Nguyen et al. 2003). The burden associated with deficits in cognitive function is expected to grow as the global population ages (Ferri et al. 2005), but few interventions to prevent, delay, or halt the progression of cognitive decline are currently available. The potential relationship between exposure to common environmental toxicants and cognition in older adults has received relatively little consideration compared with nonenvironmental risk factors, despite evidence that many toxicants may be neurotoxic and the modifiable nature of many environmental exposures.

Air pollution may exert adverse effects on central nervous system (CNS) function. In autopsy studies of dogs and people, Calderón-Garcidueñas et al. (2002, 2003, 2004, 2008b) found evidence of increased levels of inflammatory mediators, β-amyloid deposition, and markers of oxidative damage to DNA, as well as evidence of blood–brain barrier disruption, in the brains of those from a city with high levels of air pollution compared with those from a city with low levels. Children and dogs residing in a highly polluted city were also more likely to exhibit white matter hyperintensities in the prefrontal cortex than were those residing in a city with lower levels (Calderón-Garcidueñas et al. 2008a). Although this ecological evidence is insufficient to conclude that air pollution is the causal agent, other lines of evidence support this claim. In animal studies, exposure to ozone has been linked to neuroinflammation, lipid peroxidation, and memory deficits (Block and Calderón-Garcidueñas 2009; Dorado-Martinez et al. 2001), effects that may be mediated by the induction of systemic inflammation. Controlled animal experiments demonstrate that ultrafine particulate matter (PM), a component of traffic-related air pollution, can translocate to the brain via the olfactory nerve or systemic circulation (Elder et al. 2006; Oberdörster et al. 2004; Peters et al. 2006), where it may promote CNS dysfunction. In animal studies, exposure to fine or ultrafine PM has been associated with CNS inflammation and lipid peroxidation (Campbell et al. 2005; Kleinman et al. 2008; Zanchi et al. 2008), neuronal degeneration (Veronesi et al. 2005), and behavioral changes (Zanchi et al. 2008). Alternately, the cardiovascular effects of air pollution (Brook 2007; Pope and Dockery 2006) may lead to CNS dysfunction through promotion of vascular brain pathology.

One potential manifestation of CNS dysfunction in older adults is cognitive impairment. We hypothesized that exposure to black carbon (BC), a marker of traffic-related air pollution, would be associated with cognitive function in a cohort of older men, because traffic-related air pollution is a major source of exposure to ultrafine PM, which may induce CNS dysfunction.

## Materials and Methods

### Study sample

We drew the study sample from participants of the U.S. Department of Veterans Affairs (VA) Normative Aging Study (NAS), an ongoing longitudinal cohort study of men established in 1963 (Bell et al. 1972). Participants in the NAS are invited to undergo an in-person examination every 3 years. In addition to a physical examination and laboratory tests, participants provide information on medical history, lifestyle, and demographic factors at each study visit and, starting in 1993, have been asked to complete a battery of cognitive tests. At each visit, participants provided written informed consent as approved by the VA Boston Healthcare System Institutional Review Board (IRB). This study has been approved by the IRBs of the participating institutions.

We limited the study sample to participants of the NAS who completed cognitive testing between 1996 and 2007 and who lived within the range of our BC exposure model. These constraints ensured that both cognitive test data and an estimate of BC exposure before cognitive assessment were available for each study participant. We also excluded participants if they experienced a stroke before study start. In addition, we restricted the present study population to those self-reporting white race/ethnicity.

### Exposure assessment

Estimates of BC exposure at the residence of each participant were used as a surrogate for individual exposure to traffic-related air pollution. We estimated BC exposures using a validated spatiotemporal land-use regression model that, starting in 1995, provides daily estimates of BC concentrations throughout the greater Boston, Massachusetts, area, which roughly includes the area within Interstate 495; details of this model have been published previously (Gryparis et al. 2007). Briefly, daily average BC estimates from 83 monitoring sites were used to develop a BC prediction model. Predictors in the final model include information on meteorological conditions (e.g., wind speed), land use (e.g., traffic density), daily BC concentrations at a central monitor, and other descriptors (e.g., day of the week). Based on the training set, the model *R*^2^ was 0.83, whereas the average correlation between predicted values and observed BC levels in four out-of-sample validation samples was 0.59. For the present study, addresses with predicted daily BC concentrations outside the range of the exposure measurements from the training set were excluded. To create a metric of long-term exposure, we took the average of the 365 daily estimate at the participant’s residential address prior to the date of the first cognitive assessment completed on or after 1 January 1996, the first date for which computation of a prior 1-year average was possible.

### Cognitive testing

Since the onset of cognitive testing, the cognitive battery has been revised, reflecting both the addition and subtraction of individual cognitive tests. Seven cognitive tests have been administered with relative consistency across the span of cognitive testing and were considered in this study: the Mini-Mental State Examination (MMSE), the digit span backward test, a verbal fluency task, constructional praxis, immediate recall of a 10-word list, delayed recall of a 10-word list, and a pattern comparison task. The MMSE assesses multiple cognitive domains and is widely used as a dementia screening and research instrument (Tombaugh and McIntyre 1992); the six additional tests assess a variety of domains, including attention, memory, executive function, language, and visuomotor ability. All cognitive tests were drawn from established cognitive test batteries, including the Consortium to Establish a Registry for Alzheimer Disease (CERAD) battery (Morris et al. 1989), the Wechsler Adult Intelligence Scales for Adults, Revised (WAIS-R) (Wechsler 1981), the Neurobehavioral Evaluation System 2 (Letz 1991), and the Developmental Test of Visual-Motor Integration (Beery and Buktenica 1967).

The present study includes cognitive data from study visits from 1996 to 2007. Therefore, the baseline visit for each study participant is defined as the first cognitive assessment completed on or after 1 January 1996. This restriction ensures that we have an estimate of BC exposure obtained before the cognitive test data used in our analyses. Most study participants have completed multiple waves of cognitive testing, and all available cognitive data obtained between 1996 and 2007 were used in analyses.

### Statistical analysis

Because we observed a ceiling effect for MMSE scores, with many men achieving the maximum score, we created a dichotomous variable for inferior MMSE performance for use in analyses. MMSE scores are commonly used to screen for dementia (Tombaugh and McIntyre 1992), but few men in our cohort exhibited scores that would typically trigger further evaluation (< 24). We therefore chose to label scores ≤ 25 as “low” MMSE scores (18% of our observations). Cognitive test scores for the remaining six tests were *z*-transformed based on the mean and standard deviation of the individual test scores at baseline and were considered as continuous variables in analyses, with positive *z*-scores indicating better performance for all tests.

We evaluated the association between BC and the odds of having a low MMSE score using logistic regression with generalized estimating equations and empirical variance estimates to account for repeat measures across individuals. For the remaining six tests, which yield seven unique scores (the pattern comparison task produces two unique scores), we treated each cognitive test score as a repeat measure of global cognitive function in a random effects linear mixed model. This model included a random intercept for individual to account for repeat measures within individuals and a random intercept for cognitive test score to account for use of seven different cognitive test score types as repeat measures of global cognitive function. This analysis provides one hypothesis test for the association between BC and global cognitive function. We evaluated the presence of heterogeneity of BC effect across cognitive test score type by adding and testing the significance of a random slope for BC effect by cognitive test score type. If the slope were to be significant, this would indicate that the BC effect is dissimilar across cognitive test score types and the global analyses would be inappropriate. Final models were multivariable adjusted for potential confounders or predictors of cognitive function that are not plausible intermediates for a BC–cognition relationship. We obtained data on these covariates from information collected at each NAS study visit. Final multivariable-adjusted models were adjusted for age at cognitive assessment as well as information on several variables at baseline: education (< 12, 12–16, > 16 years), alcohol intake (< 2 drinks/day, ≥ 2 drinks/day), physical activity [< 12, 12 to < 30, ≥ 30 metabolic equivalent hours (MET-hr) per week], diabetes (yes/no), dark fish consumption (less than once a week, equal to or greater than once a week), computer experience (yes/no), first language (English/not English), percentage of the participant’s census tract that is nonwhite, percentage of the adult residents in the participant’s census tract with at least a college degree, an indicator for whether the cognitive data were from the participant’s first cognitive assessment (yes/no), and an indicator for whether the participant was a part-time resident of the greater Boston area (yes/no). We excluded data from study visits for which we lacked covariate information from all analyses (1.4% of study visits).

We log-transformed BC estimates for use in all analyses after examination of restricted cubic splines indicated a log-linear relationship. We additionally explored interactions between BC and body mass index (BMI), hypertension, smoking status, and diabetes using multiplicative interaction terms and conducted sensitivity analyses further adjusting our multivariable models for smoking and BMI. We used R (version 2.10.1; R Development Core Team 2010) to examine splines and SAS (version 9.2; SAS Institute Inc., Cary, NC) for all other analyses.

### Sensitivity analyses: potential confounding by lead

Exposure to lead may confound the relationship between BC and cognitive function. Traffic-related exposures were a primary source of lead exposure in the era of leaded gasoline, and it is reasonable to expect that men with high current traffic-related air pollution exposures had high past traffic-related lead exposures. In addition, cumulative past lead exposure may have an adverse impact on cognitive function in older adults (Shih et al. 2006), even at low levels (Weisskopf et al. 2007; Wright et al. 2003). Thus, we conducted sensitivity analyses to determine the influence of potential confounding by exposure to lead.

Tibia bone lead concentrations, a biomarker for cumulative past lead exposures (Hu et al. 1998), were available for 363 participants (53.4%). Tibia bone lead concentrations were imputed for 299 participants (44.0%) using a published prediction model developed in the NAS (reduced model) (Park et al. 2009). Missing data precluded imputation of bone lead concentrations for 18 participants (2.6%), and we excluded these participants from the sensitivity analyses. To test the sensitivity of our results to potential confounding by exposure to lead, we repeated the analyses described above additionally adjusting for bone lead concentration and imputation status (yes/no).

## Results

A total of 680 men met the inclusion criteria for this study. Within this group, the number of men completing at least one wave of cognitive testing varied across the cognitive tests: MMSE (*n* = 671), digit span backward test (*n* = 663), verbal fluency task (*n* = 670), constructional praxis (*n* = 626), immediate recall of a 10-word list (*n* = 669), delayed recall of a 10-word list (*n* = 667), and pattern comparison task (*n* = 645).

**Figure 1:**
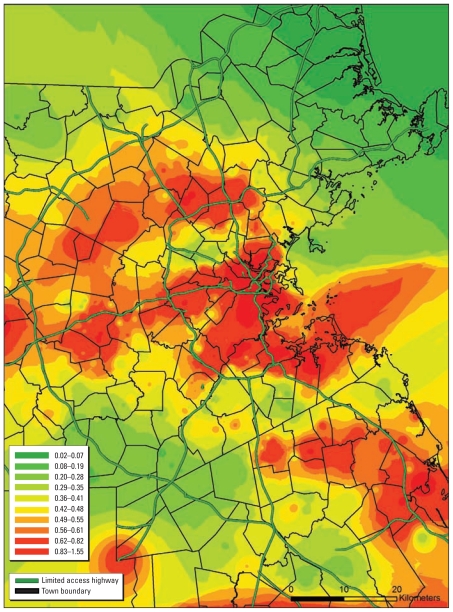
Predicted 1-year average BC exposures (μg/m^3^) for the greater Boston area in 1995.

Table 1 summarizes the baseline characteristics of the cohort. The mean ± SD age of the study sample at baseline was 71 ± 7 years (range, 51–97 years), and most men had at least some college or graduate-level education (70.1%). On average, study participants completed 2.14 cognitive assessments (range, 1–5). On the natural scale, 1-year average BC exposure estimates ranged from 0.03 to 1.77 μg/m^3^ (mean ± SD, 0.58 ± 0.28 μg/m^3^) and exhibited a skewed distribution. Figure 1 illustrates the substantial spatial variability of BC over the study area in the year 1995.

Because the association between cognitive test scores and BC exposure estimates appeared log-linear, we log-transformed BC for use in all analyses and report associations for a doubling in BC concentration on the natural scale, or approximately a 0.69 unit change in ln(BC). BC exposure was significantly associated with the risk of having a low MMSE score [multivariable-adjusted odds ratio (OR) = 1.3 for a doubling in BC concentration; 95% confidence interval (CI), 1.1–1.6; Table 2]. In the analysis of global cognitive function, which combined the remaining seven cognitive test scores, a doubling in BC was significantly associated with a decrement of 0.054 SD in cognitive test score in our multivariable-adjusted model (95% CI, −0.103 to −0.006; Table 2), an effect similar to a 1.9-year difference in age in our data. We found no evidence of heterogeneity of results across tests [*p*-value = 0.25]. See Supplemental Material, Tables 1 and 2 (doi:10.1289/ehp.1002767)] for the full multivariable-adjusted models. In our sensitivity analyses, we additionally adjusted for smoking and BMI, and our results were materially unchanged.

We found no evidence of interactions between BC and diabetes, smoking, BMI or hypertension on MMSE scores (*p*-values for all interactions > 0.1). Our exploration of potential interactions between BC and these factors on global cognitive function, in models that combined the remaining seven test scores, suggested that the adverse effects of BC were concentrated in overweight and obese individuals (*p*-value for interaction = 0.10) and in ever smokers (*p*-value for interaction = 0.07), but we found no evidence for effect modification by hypertension or diabetes (*p*-values for interactions > 0.1).

Among participants with measured tibia bone lead concentrations, the mean ± SD bone lead concentration was 19.2 ± 11.9 μg lead/g bone mineral; among those with imputed bone lead concentrations, it was 16.1 ± 7.0 μg lead/g bone mineral. In the sensitivity analysis that adjusted for estimates of cumulative lead exposure, we observed that the multivariable-adjusted association between BC and low MMSE scores was almost identical (OR = 1.3 per doubling in BC; 95% CI, 1.1–1.7). The association between BC and global cognitive function was somewhat attenuated after we adjusted for lead in our multivariable-adjusted model (−0.038 per doubling in BC; 95% CI, −0.089 to 0.012).

## Discussion

The results of this study suggest an adverse effect of traffic-related air pollution on global cognitive function in older men. Confounding by lead cannot fully account for these findings. When we explored the potential for effect modification, our results suggest that the effect of traffic-related air pollution on cognition may be greater in smokers or overweight and obese individuals. Because these conditions are proinflammatory, this is biologically plausible but requires further confirmation.

To our knowledge, this is only the second study to consider the association between air pollution and cognitive function in older adults. Ranft et al. (2009) considered the association between two markers of air pollution, a measure of PM with an aerodynamic diameter ≤ 10 μm (PM_10_) and distance to a major road, and cognitive function in 399 women 68–79 years of age who had lived at the same address for 20 years. Residence within 50 m of a major road, but not PM_10_, was significantly associated with a decrement in the total score of the CERAD-Plus test battery, the Stroop test, and a test of olfactory function in multivariable-adjusted models. In addition, residence within 50 m of a major road was adversely associated with performance on all subtests of the CERAD battery, although few individual associations achieved statistical significance. Our findings are consistent with these, because BC, a traffic particle, is strongly associated with distance to major road and weakly associated with PM_10_.

A handful of studies have evaluated the association between air pollution and cognitive function in younger populations, and, collectively, they also suggest an adverse effect. In a study of adults 20–59 years of age, Chen and Schwartz (2009) reported that estimates of exposure to ozone, but not PM_10_, were associated with inferior performance on two of three neurobehavioral tests in fully adjusted models. Calderón-Garcidueñas et al. (2008a) found the performance age on several subtests of the Wechsler Intellegence Scale for Children, Revised (WISC-R) to be significantly behind chronological age in children residing in cities with high levels of air pollution but not in those residing in cities with low levels. In a study that compared neurobehavioral test scores across students from two primary schools in the same city in China, Wang et al. (2009) found that students who attended the school with high levels of traffic-related air pollution were more likely to exhibit poor performance than were those who attended the school with low levels. Franco Suglia et al. (2008) found that higher residence-based estimates of average lifetime exposure to BC were associated with lower cognitive test scores in children 8–11 years of age. Similarly, in 4- to 5-year-old children from the INMA (Environment and Childhood) study, Freire et al. (2010) observed that higher residence-based estimates of nitric dioxide were associated with lower scores on all subscales of a standardized version of the McCarthy Scales of Children’s Abilities, although only one of these associations achieved statistical significance.

Several biological mechanisms have been proposed to explain how traffic-related air pollution may have an adverse effect on CNS function. First, ultrafine particles, such as those found in diesel exhaust, are small enough to pass through the air–blood barrier of the lung, enter the systemic circulation, and translocate to other body tissues, including the brain (Oberdörster et al. 2004; Peters et al. 2006), and may also translocate directly to the brain via the olfactory nerve (Calderón-Garcidueñas et al. 2003; Elder et al. 2006). The presence of such particles in the brain is associated with neuroinflammation and oxidative stress (Calderón-Garcidueñas et al. 2003; Elder et al. 2006). Second, traffic-related PM may exert indirect effects on CNS function through effects on cardiovascular health. Exposure to PM is associated with a variety of cardiovascular end points (Brook 2007; Pope and Dockery 2006), including hypertension (Auchincloss et al. 2008; Brook 2007) and atherosclerosis (Künzli et al. 2005, 2010), as well as markers of increased cardiovascular risk, including progression of atherosclerotic plaques (Floyd et al. 2009; Künzli et al. 2010; Sun et al. 2005) and homocysteine (Baccarelli et al. 2007; Park et al. 2008). Several of these factors are also associated with worse cognitive function (Komulainen et al. 2007; Qiu et al. 2005; Sander et al. 2010; Vidal et al. 2008), which likely reflects the link between cognitive impairment and vascular brain pathology.

The present study has several strengths. The BC exposure model enabled us to report individual estimates of long-term exposure to traffic-related air pollution. In our study, we were also able to control for socioeconomic status (SES) using both personal and residence-based characteristics, which should limit residual confounding by this factor. Furthermore, we were able to address the potential for confounding by past exposure to lead, another environmental pollutant that may affect cognitive function in older adults, using a combination of measured and imputed biomarkers for cumulative past lead exposure. Although correlated with traffic-related air pollution, exposure to lead cannot completely account for the observed associations. Finally, our results are based on a substantial number of observations, given that, on average, each of our 680 participants completed 2.14 cognitive testing sessions.

Several potential sources of bias must be considered when interpreting our results. The use of exposure estimates based on residential address may misclassify personal exposure levels. However, the lack of significant occupational or commuting exposure in this largely retired cohort should mean that residence-based exposure estimates are an excellent measure of personal exposures over the past few years. Because 87% of our participants have lived at the same address for at least 5 years before their baseline cognitive assessment (mean ± SD duration, 18 ± 12 years) and because people are likely to choose similar neighborhoods when relocating, misclassification due to participant relocation is also expected to be minimal. In addition, occasional exposures encountered elsewhere are unlikely to be correlated with cognitive function and so would result in nondifferential misclassification of exposure, which would be expected to attenuate our effect estimates (Gryparis et al. 2009). Similarly, nondifferential misclassification of cognitive function is expected when assessing cognitive function and would make it harder to detect a true effect, although our use of global analyses helps to reduce the impact of noise by using multiple measures to capture underlying global cognitive function. We observed statistically significant associations between BC and cognitive function, when measured as the risk of a “low” MMSE score and as overall performance on our battery of cognitive tests, despite the presence of misclassification, suggesting that the true association may be even stronger than what we observed. Although residual confounding cannot be excluded, we do not believe confounding to be a major source of bias in the present study given the availability of information on potential confounders and the use of several different, related variables to account for SES. Moreover, we do not believe confounding by regional pollutants, which may have an independent effect on cognition, to be a major source of bias. Correlation between BC and regional pollutants may be induced through similar spatial or temporal patterns. However, traffic-related air pollution exhibits significant spatial variation not shared by more regional pollutants [e.g., ozone or particulate matter with an aerodynamic diameter < 2.5 μm (PM_2.5_)], and temporal correlation is quite weak between our 1-year residence-based BC estimates (which have varying start dates depending on the timing of the baseline cognitive assessment) and the 1-year average of ozone concentrations at four central monitors in the Boston area (*r* = −0.03) or 1-year average PM_2.5_ concentrations at a central site monitor in Boston (*r* = 0.16) calculated over the same time periods. Incomplete follow-up may induce bias in the present study but is unlikely to account for the observed associations. In our study, and generally in studies of older adults, participants who are lost to follow-up exhibit lower cognitive test scores than do those who complete follow-up (Euser et al. 2008). Because air pollution has been related to cardiovascular morbidity and mortality (Brook 2007; Pope and Dockery 2006) and because we observed lower BC estimates in participants with three or more follow-up visits, we expect that a greater loss of those with both lower cognitive test scores and higher BC estimates to lead to an underestimate of the association. Thus, the true association may be stronger than that observed.

One additional limitation lies in our inability to attribute our findings to a particular traffic-related exposure. We have estimated exposure to BC particles for each participant. However, traffic-related air pollution is a complex mixture of gases and particles, and BC is correlated with other components of the mixture. Although existing evidence suggests that PM, specifically ultrafine PM, is a likely agent for adverse effects to the CNS (Calderón-Garcidueñas et al. 2003; Elder et al. 2006; Oberdörster et al. 2004; Peters et al. 2006), other components may be important. In addition, measures of traffic-related air pollution are often correlated with other environmental exposures. Although we were able to address the potential for lead to account for the observed associations, other exposures must be considered, including noise. Elevated noise levels have been linked to metrics of cognitive function in children in a handful of studies (Clark et al. 2006; Stansfeld et al. 2005), and chronic occupational exposure to noise is associated with neurophysiological measures related to attention in adults (Gomes et al. 1999). Given the high correlation between measures of traffic-related air pollution and noise (Allen et al. 2009; Davies et al. 2009), it is possible that the associations observed for one exposure are attributable to the other. Further research to evaluate the relative contribution of particular traffic-related pollutants or other exposures to the observed association is warranted.

## Conclusions

Our findings suggest that traffic-related air pollution may have an adverse effect on cognition in older men. This is the first study to find an association between traffic-related air pollution and cognition in older men, and only the second to consider the relationship in older adults. Given the ubiquitous nature of the exposure, if traffic-related air pollution is causally related to cognitive impairment in older adults, implementation of interventions to reduce exposure, including establishment of more stringent emissions standards, would be expected to have substantial benefits.

## Figures and Tables

**Table 1:** Baseline characteristics of the cohort (*n* = 680).

Characteristic	*n* (%)	BC concntration (mean ± SD)
Age (years)		
50–59	48 (7.1)	0.550 ± 0.288
60–69	284 (41.8)	0.574 ± 0.277
70–79	273 (40.1)	0.583 ± 0.267
80–89	72 (10.6)	0.605 ± 0.333
90–99	3 (0.4)	0.466 ± 0.285

Education (years)		
< 12	203 (29.9)	0.629 ± 0.304
12–16	338 (49.7)	0.572 ± 0.276
> 16	139 (20.4)	0.521 ± 0.239

First language		
English	585 (86.0)	0.578 ± 0.282
Not English/bilingual	95 (14.0)	0.581 ± 0.266

Computer experience		
Yes	277 (40.7)	0.542 ± 0.255
No	403 (59.3)	0.604 ± 0.294

Physical activity (MET-hr/week)		
< 12	385 (56.6)	0.574 ± 0.286
12 to < 30	178 (26.2)	0.602 ± 0.293
≥ 30	117 (17.2)	0.558 ± 0.237

Alcohol (drinks/day)		
≥ 2	166 (24.4)	0.573 ± 0.242
< 2	514 (75.6)	0.581 ± 0.291

Diabetes		
Yes	106 (15.6)	0.567 ± 0.254
No	574 (84.4)	0.581 ± 0.285

Consumed dark fish (times/week)		
≥ 1	100 (14.7)	0.566 ± 0.283
< 1	580 (85.3)	0.581 ± 0.280

Nonwhite (% of census tract)		
< 5%	268 (39.4)	0.521 ± 0.298
5 to < 10%	188 (27.6)	0.541 ± 0.249
≥ 10%	224 (32.9)	0.679 ± 0.255

≥ 25 years of age with at least a college degree (% of census tract)		
< 30%	214 (31.5)	0.626 ± 0.301
30 to < 50%	281 (41.3)	0.540 ± 0.277
≥ 50%	185 (27.2)	0.583 ± 0.250

Smoking		
Never	192 (28.2)	0.557 ± 0.255
Former	453 (66.6)	0.584 ± 0.294
Current	35 (5.1)	0.634 ± 0.208

BMI (kg/m^2^)		
< 25	152 (22.4)	0.587 ± 0.262
≥ 25	528 (77.6)	0.576 ± 0.285

Hypertension		
Yes	454 (66.8)	0.587 ± 0.288
No	226 (33.2)	0.562 ± 0.264

Tibia bone lead		
Measured	363 (53.4)	0.599 ± 0.286
Imputed	299 (44.0)	0.559 ± 0.271
Missing	18 (2.6)	0.507 ± 0.289

**Table 2:** Adjusted associations for a doubling in BC concentration on the natural scale and cognitive test score(s).

	Effect per doubling in BC concentration (95% CI)		
Analysis	Model 1: age adjusted	Model 2: age and education adjusted	Model 3: multivariable adjusted^a^
Low MMSE score OR	1.4 (1.1 to 1.6)	1.3 (1.0 to 1.5)	1.3 (1.1 to 1.6)
Global analysis estimate	−0.073 (−0.122 to −0.023)	−0.052 (−0.100 to −0.004)	−0.054 (−0.103 to −0.006)

a Adjusted for age, education, first language, computer experience, physical activity, alcohol consumption, diabetes, dark fish consumption, percentage of residential census tract that is nonwhite, percentage of residential census tract adults with a college degree, indicator for first cognitive assessment, and indicator for part-time resident.
